# Medical Students and Their Perceptions of Digital Medicine: a Question of Gender?

**DOI:** 10.1007/s40670-022-01594-x

**Published:** 2022-08-02

**Authors:** Valentina Faihs, Christina Figalist, Eileen Bossert, Katja Weimann, Pascal O. Berberat, Marjo Wijnen-Meijer

**Affiliations:** 1 TUM Medical Education Center, TUM School of Medicine, Technical University of Munich, Ismaninger Str. 22, 81675 Munich, Germany; 2 Department of Dermatology and Allergy Biederstein, TUM School of Medicine, Technical University of Munich, Biedersteiner Str. 29, 80802 Munich, Germany

**Keywords:** Medical education, Digital medicine, Digital literacy, Curriculum, Wearables

## Abstract

Digital technologies play an essential role in the medical sector of today and the future. In a cross-sectional online survey at a German medical university, male students more frequently reported keeping themselves informed about digital medicine outside of their studies across all clinical years of study. While female students self-assessed their knowledge in different fields of digital medicine as worse than their male peers in the first clinical years of study, no more gender differences could be found towards the final year. However, students of both genders showed a strong desire for further education on the topic of digital medicine.

## Background

Digital technologies are an essential part of today’s medicine and will continue to play an increasingly important role in the future. Expanding digitalization is expected to provide better patient care through more precise, personalized, and accessible medicine at higher efficiency and lower costs [1–6]. Digital medicine should not replace the personal interaction with patients, but rather reinforce it by eliminating repetitive work and the use of digital communication methods [7–9]. In this digital transformation process, however, also new challenges arise [1, 4, 10, 11]. Current and future healthcare professionals play a central role in this digitalization process, making it essential to address new digital technologies and digital literacy in medical school [12–15].

At individual universities, teaching projects about digital medicine have been very well received by students [16–18]. However, a comprehensive thematization of the new technological possibilities as well as the accompanying challenges in medical studies has been lacking so far, although this is increasingly demanded [13, 15, 19]. Recently, a study showed that medical students across Europe perceived a lack of digital health literacy [20].

A study at an Austrian medical university found that female medical students reported a significantly worse knowledge regarding medical information and communication technologies as well as telemedicine [21]. Additionally, social psychological models have previously suggested that men are less fearful of using mobile technologies than women and show a greater willingness to use them in the medical context [22]. Gender differences have been described in multiple aspects of physicians’ daily life [23–26]. Currently, the issue of gender sensitivity in teaching gets more and more attention [27–32]. However, to our knowledge, potential gender differences among medical students regarding digital medicine and their desire for further training in this field have not yet been investigated. As digital medicine is the future, insights into possible gender-related differences in this field are indispensable. This knowledge should help include teaching on digital medicine in the medical curriculum in the best possible way to best prepare all future physicians for the ongoing digitalization in the medical field.

## Activity

We performed a questionnaire-based, cross-sectional, explorative study among medical students in the clinical semesters at the Technical University of Munich (TUM). It was developed by members of the TUM Medical Education Center in a multi-step process to obtain face validity with the aim to help assess the students’ needs and wishes regarding digital health literacy, as an elective course to this topic should be established. The questionnaire was created using the software EvaSys (Evaluationssysteme GmbH, Lueneburg, Germany) and was available online from July 30 to August 17, 2020. All students in the clinical semesters (corresponding to the last 4 years of the 6-year study course in Germany) at TUM were invited to participate by e-mail and reminded once.

In the anonymous survey, participants were asked about their gender, age, and clinical year of study (1st, 2nd, 3rd, or final year) in German. In addition, students were asked about their opinions, self-assessment, and desire for further education regarding different topics of digital medicine using 5-point Likert-type scales as well as organizational preferences for the planned elective course (data not shown).

Statistical analysis was performed using SPSS Statistics 27.0 (SPSS Inc., Chicago, USA). Differences were calculated using Mann–Whitney *U* tests. Cronbach’s alpha was calculated for reliability analysis. Data are given as means and standard deviations (SD) or medians and interquartile range (IQR). A *p*-value of < 0.05 was considered statistically significant. This study was conducted in accordance with the ethical criteria of the Declaration of Helsinki.

## Results

Out of 1574 medical students invited to participate, 218 students completed the survey (response rate 13.9%). Of these, 139 identified themselves as female (63.8%), 78 as male (35.8%), and 1 as diverse (0.5%), corresponding to the general gender distribution among medical students at TUM. Mean age was 24.7 years (SD 3.5 years), and students from all clinical years participated (23.4% in the 1st, 30.3% in the 2nd, 22.5% in the 3rd, and 22.9% in the final year).

Table 1 shows the gender-specific responses of the medical students about their opinions on the topic of digital technologies in medicine. Most students of both genders equally believed that medicine will be fundamentally changed by new digital opportunities in the next few years. More than a quarter of the students rather or fully agreed with the statement that they fear the digital challenges in the medical profession. Male students found it more important to be able to question the results of innovative digital technologies (*p* < 0.01). While 21.8% of the male students fully or rather agree that they feel well prepared for the digital challenges in their future profession, this was the case for just 11.5% of female students (*p* < 0.05). Men were also significantly more likely to report to keep themselves informed about digital medicine outside of their studies (*p* < 0.001) and to find it important to be informed about the current possibilities and perspectives of digital medicine (*p* < 0.05). However, most students wanted to use innovative digital technologies in their future medical profession, with a higher degree of approval among male students (*p* < 0.05).

**Table 1:** Opinions regarding digital technologies in medicine, stratified by gender

	***I do not agree* = *1***	***2***	***3***	***4***	***I fully agree* = *5***	***p***	***Mean***	***Median***	***SD***	***IQR***
**I believe that new digital technologies will fundamentally change medicine in the next few years**										
m	2.6%	7.7%	12.8%	32.1%	44.9%	0.195	4.09	4	1.06	1.0
f	0.7%	3.0%	10.8%	33.8%	51.1%		4.31	5	0.86	1.0
**I fear the digital challenges in the medical profession, e.g., regarding data protection**										
m	19.7%	40.8%	14.5%	13.2%	11.8%	0.068	2.57	2	1.28	1.8
f	15.1%	27.3%	22.3%	27.3%	7.9%		286	3	1.21	2.0
**To me, it is important to be able to question the results of innovative digital technologies**										
m	1.3%	3.9%	7.8%	26.0%	61.0%	0.004^**^	4.42	5	0.89	1.0
f	2.2%	5.8%	17.3%	33.8%	41.0%		4.06	4	1.01	2.0
**Overall, I feel well prepared for the digital challenges I will face in the medical profession**										
m	21.8%	19.2%	37.2%	12.8%	9.0%	0.020^*^	2.68	3	1.21	1.0
f	16.7%	48.6%	23.2%	10.1%	1.4%		2.31	2	0.92	1.0
**To me, it is important to be informed about the current possibilities and perspectives of digital medicine**										
m	1.3%	2.6%	3.8%	32.1%	60.3%	0.022^*^	4.47	5	0.80	1.0
f	0.7%	2.2%	13.0%	39.1%	44.9%		4.25	4	0.82	1.0
**Outside of my studies, I keep myself informed about digital medicine**										
m	12.8%	25.6%	20.5%	20.5%	20.5%	< 0.001^***^	3.10	3	1.34	2.0
f	35.3%	28.1%	21.6%	10.8%	4.3%		2.21	2	1.16	2.0
**I want to actively use innovative digital technology in my future medical profession**										
m	3.8%	0.0%	11.5%	33.3%	51.3%	0.021^*^	4.28	5	0.95	1.0
f	0.7%	2.2%	20.9%	41.7%	34.5%		4.07	4	0.84	1.0

Difference between genders: Mann–Whitney *U* tests

*m* male, *f* female, *SD* standard deviation, *IQR* interquartile range

^*^p < 0.05; ^**^p < 0.01; ^***^p < 0.001

Table 2 shows the responses on self-assessed knowledge and desire for further education about digital technologies in medicine. Both subscales showed good internal consistency (Cronbach’s alpha for self-assessment = 0.804, for the desire for further education = 0.843). While 38.5% of male students self-reported their knowledge about medical wearables and apps as very good or good, this was the case for 10.8% of females (overall *p* < 0.001). Significant differences were also found regarding digital communication methods (*p* < 0.05), robotics (*p* < 0.01), and digital processes in patient management (*p* < 0.05). Most students, irrespective of gender, indicated a strong desire for further education in the context of their studies in all the surveyed areas of digital medicine.

**Table 2:** Self-assessment and desire for further education regarding digital technologies in medicine, stratified by gender

**I assess my knowledge of … in medicine as follows**										
	***Very bad* = *1***	***2***	***3***	***4***	***Very good* = *5***	***p***	***Mean***	***Median***	***SD***	***IQR***
**Artificial intelligence**										
m	12.8%	33.3%	29.5%	17.9%	6.4%	0.201	2.72	3	1.10	1.3
f	12.9%	39.6%	33.1%	12.9%	1.4%		2.50	2	0.93	1.0
**Information management**										
m	9.0%	29.5%	34.6%	17.9%	9.0%	0.060	2.88	3	1.09	2.0
f	12.9%	36.0%	34.5%	11.5%	5.0%		2.60	3	1.02	1.0
**Digital communication methods**										
m	7.7%	24.4%	20.5%	34.6%	12.8%	0.028^*^	3.21	3	1.18	2.0
f	10.1%	29.5%	30.9%	23.7%	5.8%		2.86	3	1.07	2.0
**Data protection and IT security**										
m	23.1%	34.6%	24.4%	6.4%	11.5%	0.153	2.49	2	1.25	1.0
f	25.9%	40.3%	25.2%	6.5%	2.2%		2.19	2	0.97	2.0
**Wearables and apps**										
m	10.3%	23.1%	28.2%	29.5%	9.0%	< 0.001^***^	3.04	3	1.14	2.0
f	28.1%	33.8%	27.3%	10.1%	0.7%		2.22	2	0.99	2.0
**Robotics**										
m	17.9%	28.2%	21.8%	29.5%	2.6%	0.002^**^	2.71	3	1.15	2.0
f	30.2%	31.7%	25.2%	12.9%	0.0%		2.21	2	1.02	2.0

	***I do not agree* = *1***	***2***	***3***	***4***	***I fully agree* = *5***	***p***	***Mean***	***Median***	***SD***	***IQR***
**I feel sufficiently informed about ethical issues regarding the digitalization of medicine**										
m	11.5%	34.6%	26.9%	16.7%	10.3%	0.280	2.79	3	1.17	2.0
f	18.2%	32.1%	24.8%	21.9%	2.9%		2.59	2	1.11	1.5
**I have sufficient knowledge about digital processes in patient management**										
m	20.5%	33.3%	29.5%	12.8%	3.8%	0.047^*^	2.46	2	1.08	1.0
f	29.5%	33.1%	31.7%	5.8%	0.0%		2.14	2	0.91	2.0
**In my studies, I would like to learn more about the use of artificial intelligence in medicine**										
m	1.3%	3.9%	11.7%	29.9%	53.2%	0.133	4.30	5	0.92	1.0
f	0.7%	7.2%	10.1%	41.3%	40.6%		4.14	4	0.92	1.0
**In my studies, I would like to learn more about wearables and apps in medicine**										
m	6.5%	7.8%	15.6%	32.5%	37.7%	0.509	3.87	4	1.20	2.0
f	1.4%	10.1%	18.1%	42.0%	28.3%		3.86	4	0.99	2.0
**In my studies, I would like to learn more about information management**										
m	2.6%	5.3%	19.7%	35.5%	36.8%	0.681	3.99	4	1.01	2.0
f	0.7%	7.3%	17.5%	43.8%	30.7%		3.96	4	0.92	2.0
**In my studies, I would like to learn more about digital communication methods**										
m	7.8%	7.8%	16.9%	37.7%	29.9%	0.379	3.74	4	1.20	2.0
f	2.9%	10.1%	10.1%	46.4%	30.4%		3.91	4	1.04	1.0
**In my studies, I would like to learn more about data protection and IT security**										
m	3.9%	16.9%	22.1%	23.4%	33.8%	0.539	3.66	4	1.22	2.0
f	5.1%	8.0%	21.0%	35.5%	30.4%		3,78	4	1,12	2,0
**In my studies, I would like to learn more about the use of robotics in medicine**										
m	2.6%	9.2%	9.2%	26.3%	52.6%	0.130	4.17	5	1.10	1.0
f	1.4%	8.0%	19.6%	30.4%	40.6%		4.01	4	1.03	2.0
**In my studies, I would like to learn more about ethical aspects of the digitalization of medicine**										
m	7.8%	9.1%	14.3%	32.5%	36.4%	0.498	3.81	4	1.25	2.0
f	2.90%	10.1%	16.7%	29.7%	40.6%		3.95	4	1.12	2.0

Difference between genders: Mann–Whitney *U* tests

*m* male, *f* female, *SD* standard deviation, *IQR* interquartile range

^*^p < 0.05’ ^**^p < 0.01; ^***^p < 0.001

**Fig. 1:**
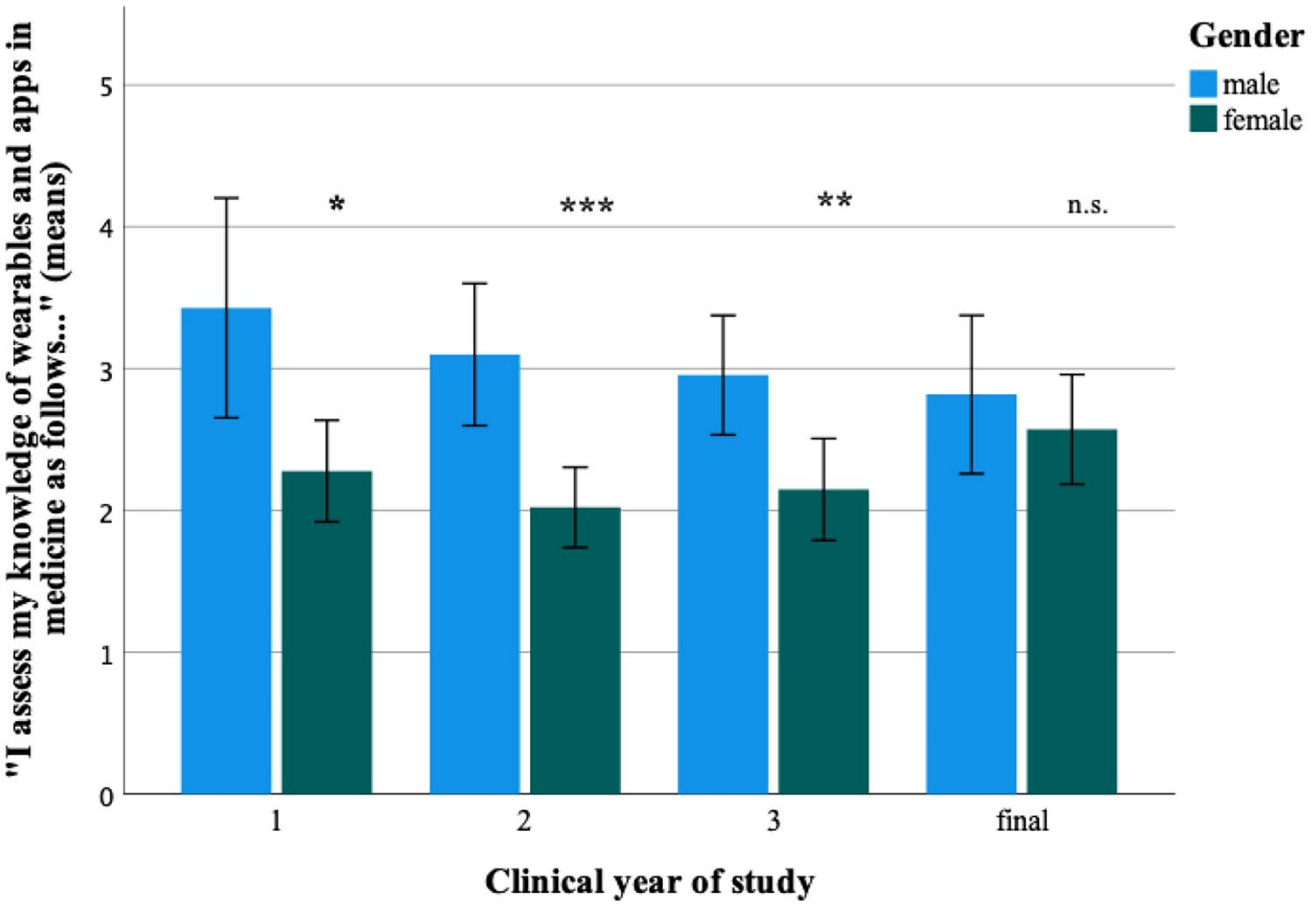
Response to the item “I assess my knowledge of wearables and apps in medicine as follows” stratified by gender and clinical year of study. Data are shown as means and 95% confidence intervals. Difference between clinical years of study: Mann–Whitney *U* tests, *p < 0.05, **p < 0.01, ***p < 0.001, n.s., difference not significant

Interestingly, subjective differences to prior knowledge were most evident in the first clinical years of study. Female students in the first year estimated their knowledge on information management (*p* < 0.01), digital communication methods (*p* < 0.05), and data protection and IT security in medicine (*p* < 0.01) to be significantly worse than their male peers, while no significant differences were seen in the third and final year. Regarding the topic of wearables and apps, a significant difference was seen only in the first 3 clinical years of study and not in the final year (Fig. 1). Across all clinical years of study, male students more frequently reported keeping themselves informed about digital medicine outside of their studies.

## Discussion

Digital technologies are playing an increasingly important role in the medical sector, so competencies in this area should be addressed as early as possible [12–15]. In today’s medical education, increasing efforts are made to make medical teaching more gender-sensitive [27–32]. To our knowledge, this is the first study to examine gender differences in medical students regarding their opinions, self-assessment, and desire for further education on specific topics related to digital medicine.

In our survey, we found some significant gender differences when medical students were asked about their opinions and perceptions of digital medicine. Unlike previous studies [22], we did not find any difference regarding the fear of digital challenges in our survey. Most students showed a great motivation to use innovative digital medicine in their future medical profession, however with higher approval rates among male students.

We found that, overall, male students indicated a higher self-assessed knowledge regarding various topics of digital medicine (digital communication methods, wearables and apps, robotics and digital processes in patient management), especially in the first clinical years of study. Our results match a survey among students and staff at an Austrian medical university, in which men rated their knowledge about electronic information and communication technologies in medicine and telemedicine better than women [21]. Several studies have already described that female medical students as well as female physicians assessed themselves significantly worse regarding practical skills than their male peers, although there seems to be no difference regarding their objective performance [33–35].

Male students more frequently reported keeping themselves informed about digital medicine outside of their studies across all clinical years of study. Interestingly, female medical students assessed their knowledge of different areas of digital medicine worse than male students especially in the early clinical semesters, whereas the differences were no longer significant in the final year. More research is needed to investigate whether the described gender differences are also evident in objectifiable knowledge and application in the field of digital medicine.

One explanation for this observation could be that differences in prior knowledge are successively compensated for by an increasing transfer of relevant knowledge during the study period. Thus, an increased and early integration of digital medicine topics into the medical curriculum could potentially compensate for existing differences early in the studies and prepare both future female and male physicians for their professional lives in the best possible way. Another explanation could be an increase in self-confidence of female students during the study course, compensating for a possible bias in self-assessment. Previous studies have already described that female medical students self-assessed their practical skills as worse than their male peers even though no difference in terms of objective performance could be found [36, 37]. Further studies are needed to better elucidate these topics.

However, we found a strong desire for further education on all surveyed topics irrespective of gender. These results are consistent with a Europe-wide study in which medical students expressed a desire for a stronger thematization of digital medicine in their studies [20].

Of course, this study has some limitations. Due to the monocentric character of this study, results cannot be generalized. Biases due to the online character, the limited time availability of the survey, or the low response rate cannot be ruled out either. Moreover, this cross-sectional study does not allow any conclusions about possible causalities.

## Data Availability

The datasets generated during and/or analyzed during the current study are available from the corresponding author on reasonable request.
